# Optimization and expansion of non-negative matrix factorization

**DOI:** 10.1186/s12859-019-3312-5

**Published:** 2020-01-06

**Authors:** Xihui Lin, Paul C. Boutros

**Affiliations:** 1 Informatics & Biocomputing, Ontario Institute for Cancer Research, Toronto, Canada; 2 Department of Human Genetics, University of California, Los Angeles, USA; 3 Jonsson Comprehensive Cancer Center, University of California, Los Angeles, USA

**Keywords:** Non-negative matrix factorization, Deconvolution, Imputation

## Abstract

**Background:**

Non-negative matrix factorization (NMF) is a technique widely used in various fields, including artificial intelligence (AI), signal processing and bioinformatics. However existing algorithms and R packages cannot be applied to large matrices due to their slow convergence or to matrices with missing entries. Besides, most NMF research focuses only on blind decompositions: decomposition without utilizing prior knowledge. Finally, the lack of well-validated methodology for choosing the rank hyperparameters also raises concern on derived results.

**Results:**

We adopt the idea of sequential coordinate-wise descent to NMF to increase the convergence rate. We demonstrate that NMF can handle missing values naturally and this property leads to a novel method to determine the rank hyperparameter. Further, we demonstrate some novel applications of NMF and show how to use masking to inject prior knowledge and desirable properties to achieve a more meaningful decomposition.

**Conclusions:**

We show through complexity analysis and experiments that our implementation converges faster than well-known methods. We also show that using NMF for tumour content deconvolution can achieve results similar to existing methods like ISOpure. Our proposed missing value imputation is more accurate than conventional methods like multiple imputation and comparable to missForest while achieving significantly better computational efficiency. Finally, we argue that the suggested rank tuning method based on missing value imputation is theoretically superior to existing methods. All algorithms are implemented in the R package NNLM, which is freely available on CRAN and Github.

## Background

Non-negative matrix factorization (NMF or NNMF) [1] has been widely used as a general method for dimensional reduction and feature extraction on non-negative data. The main difference between NMF and other factorization methods, such as SVD, is the nonnegativity, which allows only additive combinations of intrinsic ‘parts’, i.e. the hidden features. This is demonstrated in [1], where NMF learns parts of faces and a face is naturally represented as an additive linear combination of different parts. Indeed, negative combinations are not as intuitive or natural as positive combinations.

In bioinformatics, NMF is sometimes used to find ‘metagenes’ from expression profiles, which may be related to some biological pathways [2, 3]. NMF has been used to extract trinucleotide mutational signatures from mutations found in cancer genomic sequences and it was suggested that the trinucleotide profile of each cancer type is a positive linear combination of these signatures [4].

There are several different algorithms available for NMF decomposition, including the multiplicative algorithms proposed in [1], gradient descent and alternating non-negative least square (ANLS). ANLS is gaining attention due to its guarantee to converge to a stationary point and being a faster algorithm for non-negative least squares (NNLS).

In this paper, we first unify various regularization forms on the result components, which encourage desired properties such as orthogonality and sparsity and show how the conventional multiplicative algorithms [1] can be modified to adapt to these regularizations inspired by [3]. We then adopt the ANLS approach, but incorporate a solution to the NNLS problem using a coordinate-wise algorithm proposed by [5], in which each unknown variable can be solved sequentially and explicitly as simple quadratic optimization problems. We demonstrate that this algorithm can converge much faster than traditional multiplicative algorithms. For NMF with Kullback-Leibler divergence loss, we extend this methodology by approaching the loss with a quadratic function.

NMF is a dimension reduction method, as the resulting decomposed matrices have a smaller number of entries than the original matrix. This means that one does not need all the entries of the original matrix to perform a decomposition, thus NMF should be able to handle missing entries in the target matrix. Indeed, factorization can be fulfilled by dropping the loss items related to the missing entries if the target loss function is a sum of per-entry losses, e.g., mean square error (MSE) or Kullback-Leibler (KL) divergence. Furthermore, the reconstructed matrix has values on entries that are missing in the original matrix. This reveals the capability of NMF for missing value imputation. Inspired by this observation and the popular training-validation tuning strategy in supervised models, we introduce a novel method to optimize the only hyper-parameter *k*, i.e. the rank of NMF decomposition.

NMF is essentially unsupervised. It performs a blind decomposition, which puts the meaning of the result in question. This might limit the applications of unsupervised methods in areas where strong interpretability is critical, including most biomedical research. On the other hand, decomposition without utilizing known discovery (prior knowledge) may not be effective, especially with a small sample size. To overcome these challenges, we apply a masking technique to the NMF decomposition during the iterating algorithms to retain certain structures or patterns in one or both resulting matrices, which can be designed according to our prior knowledge or research interest. This technique can be used to perform a pathway or sub-network guided decomposition or to separate different cell types from mixed tissue samples.

All of these algorithmic innovations are implemented in the popular R programming language. They serve as an alternative to the widely-used *NMF* package [6] which was first translated from a MATLAB package and later optimized via C++ for some algorithms. The sparse alternating NNLS (ANLS) by [3] is anticipated to be fast in theory, but its implementation in R leads to slow performance in practice. Our NNLM package combines the efficient NNLS algorithm with the use of Rcpp, which seamlessly integrates R and C++ [7] and is freely available and open-source.

In summary, the main contributions of this work include:

- unifying various type of regularizations and deriving the correspondent multiplicative algorithms;
- developing a faster algorithm for NMF using sequential coordinate-wise descent;
- introducing a method to handle missing entries in the target matrix, which results in a novel method to determine the rank *k* and a new application of NMF for missing value imputation;
- introducing a masking technique to integrate prior knowledge and desirable properties and demonstrating how it can be used to achieve tumour content deconvolution.

## Results

### Algorithms comparison

**Fig. 1:**
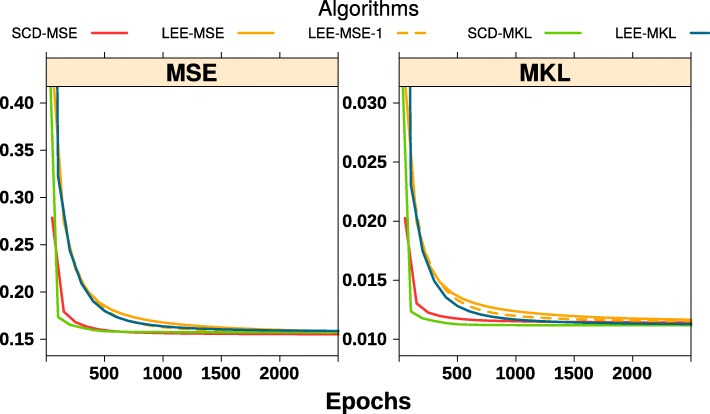
Comparison of different algorithms in convergence

We carry out an experiment for illustration purpose using a subset of microarray data from a group of Non-Small Cell Lung Cancer (NSCLC) data ([8], available in package NNLM) to compare SCD with Lee’s multiplicative algorithms. Results are shown in Fig. 1 and Table 1. Here one can see that the SCD and Lee’s algorithms have roughly the same run time for each epoch, i.e., updating *W* and *H* entries once. However, SCD generally converges much faster, achieving the same accuracy in fewer epochs and a much shorter time. Obviously, algorithms with mean KL loss are slower than those with MSE for each epoch, but reducing error a bit more in each epoch. The multiplicative algorithm with MSE is faster when a multiple-epochs update (*N*_*i*_>1) is performed in each outer alternating iteration (LEE-MSE vs LEE-MSE-1).

**Table 1:** Comparing performance of different algorithms on a subset of a non-small cell lung cancer dataset, with *k*=15

	SCD-MSE	LEE-MSE	LEE-MSE-1	SCD-MKL	LEE-MKL
MSE	0.155	0.1565	0.1557	0.1574	0.1579
MKL	0.01141	0.01149	0.01145	0.01119	0.01122
Rel. tol.	1.325e-05	0.0001381	0.000129	6.452e-08	9.739e-05
Total epochs	5000	5000	5000	5000	5000
Time (Sec.)	1.305	1.35	8.456	49.17	41.11

MSE = mean square error; MKL = mean KL divergence; Rel. tol. = relative tolerance. Elapsed time = actual running time. SCD-MSE = SCD algorithm with MSE loss and 50 inner iterations and LEE-MSE-1 = Lee’s algorithm with MSE loss and 1 inner iteration, i.e., the original multiplicative algorithm

### Missing value imputation

**Fig. 2:**
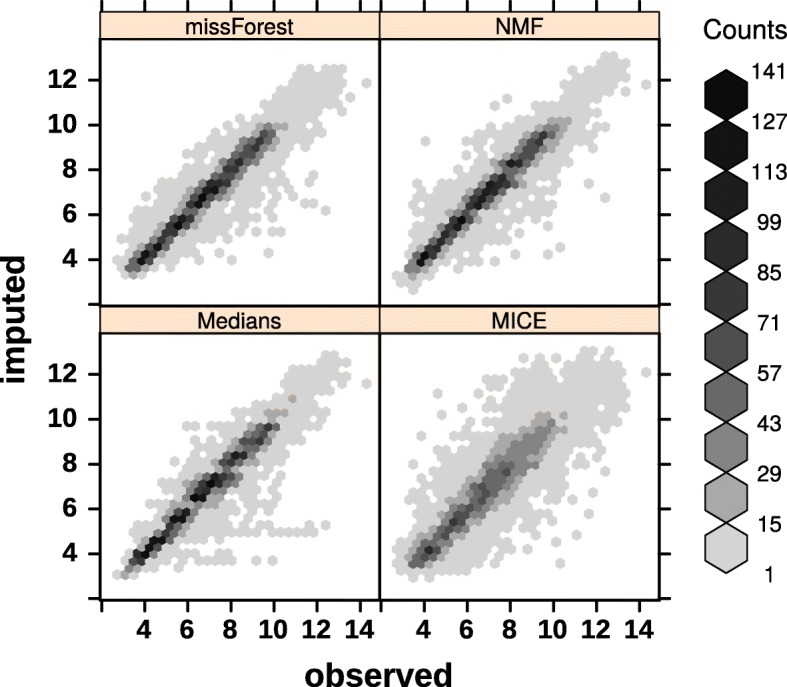
Comparison of imputation methods. *k*=2 is used for NMF

A comparison of different imputation methods are shown in Fig. 2 and Table 2. A subset of the NSCLC dataset [8] is used with 30% randomly selected to be missing. One can see that NMF is almost as good as missForest [9] but much faster, and clearly better than MICE [10] and simple median imputation in this example.

**Table 2:** A comparison of different imputation methods

	Baseline	Medians	MICE	MissForest	NMF
MSE	4.4272	0.5229	0.9950	0.4175	0.4191
MKL	0.3166	0.0389	0.0688	0.0298	0.0301
Time (Sec.)	0.0000	0.0000	90.2670	42.4010	0.1400

Imputations on a subset of NSCLC microarray data, which composes 200 genes and 100 samples. 30% of the entries are randomly deleted, i.e., missed. MSE = mean square error, MKL = Mean KL-divergence distance and Time = user time

### Choice of *k*

We performed a simulation study to illustrate our proposed method for selecting the rank hyperparameter *k*. Entries of $W \in \mathbb {R}^{400 \times 3} $ and $H \in \mathbb {R}^{3\times 50}$ are sampled independently and uniformly from interval (0,1) and (0,10) respectively. *A* was constructed by *WH* plus noise sampled independently for each entry from the standard normal distribution. All negative values in *A* are set to 0 (very unlikely). We choose MSE as loss and run the purposed algorithm 5 times, each with a random 30% entries deleted.

**Fig. 3:**
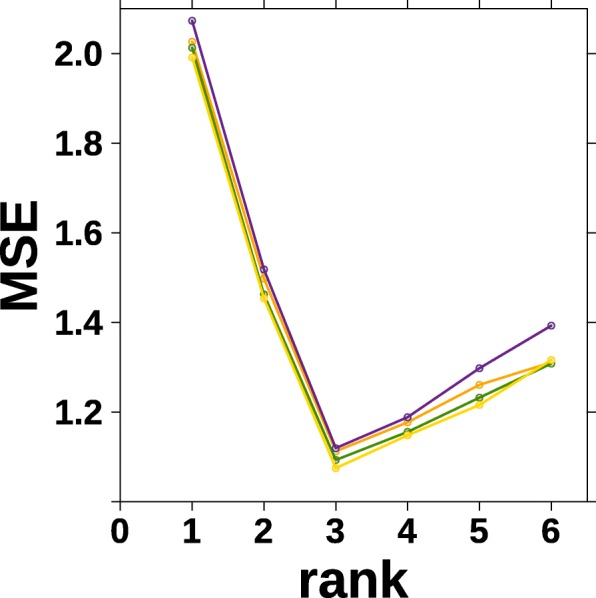
Determine optimal rank *k* in NMF using imputation

The result is shown in Fig. 3. As we could see, different runs (indicated by different colors) give consistent results. The mean square errors (MSEs) of the reconstruction of the missing entries are minimized at *k*=3 for all runs.

### Tumour content devolution

Expression deconvolution is of constant interest in bioinformatics and clinical research [11, 12]. Some NMF related methods were proposed [13]. However, our unique methods of using mask matrices are more flexible and powerful, as one can almost guide the decomposition towards any biological procedure of interest by integrating prior knowledge into the initial and mask matrices. As compared to Bayesian methods like the ISOpure [14], NMF based methods are much faster.

**Fig. 4:**
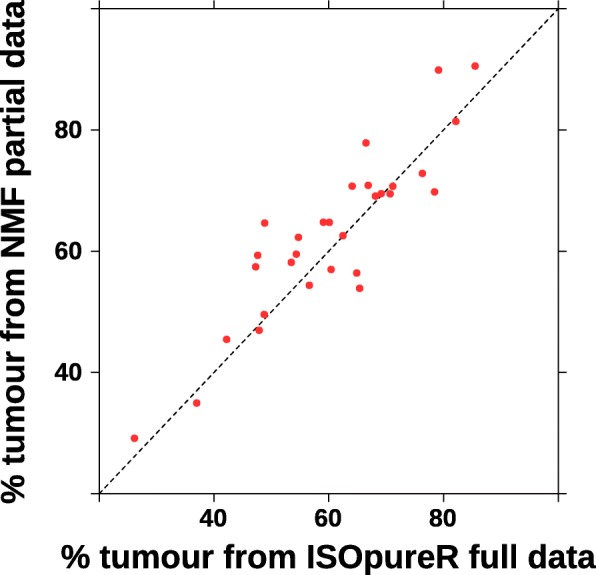
Comparing NMF to ISOpure [16] for tumour content deconvolution

We use part of Beer’s lung adenocarcinoma data[15], which contains 30 tumours and 10 normal samples, with 250 transcripts, available in the Isopure R package [16]. A comparison to the result from ISOpure using the full dataset (80 tumours and 5151 transcripts) is shown in Fig. 4. We can see that our result based on a small part of the dataset produces a comparable result.

## Discussion

We combine common regularizations of NMF into a general form to explore the power of mixing different types of regularizations. The choice of weights of the regularization should depend on the problem. For example, independence or orthogonality (*J*_2_) may be favored for content deconvolution, while sparsity (*J*_3_) might be more important for metagenes or sub-networks discovery. *J*_1_ can be used to reduce variance in the outcome matrices used for downstream analysis such as prognostic biomarker discovery. Another way to choose these hyperparameters is to use the same approach as introduced in “Choice of *k*” section for tuning *k*, i.e., choose the ones that minimize reconstruction error or variation. This can be done together with the choice of *k*.

The choice of MSE or KL as the loss function depends on the nature and the distribution of the entries. A general principle is to use MSE when the distribution of the entries are centered around a certain region, i.e., the magnitudes are roughly the same (e.g., the simulation study in “Choice of *k*” section). However, for very skewed distributions (e.g, count data) or data with outliers, the KL loss may fit better, as if MSE is used in this case, the large entries might dominate the loss while small entries have little impact, resulting in a factorization with large variance. For the latter case, one can also perform the decomposition in the log space if all entries have values greater than 1 or in the log(1+*A*) space with MSE. However, the interpretation of the results has to be changed as well.

Although NMF can be done with missing entries, when the missing process is correlated with the value itself, i.e., not missing completely at random (MCAR), the resulting reconstruction may be biased. Besides, when there are many missings, especially when a certain row or column is largely missing, the composition and the reconstruction could have a large variation and thus not reliable. The same argument also carries to the proposed method for choosing *k*.

The masking technique is simple yet useful for many applications. Here we only demonstrate its application to tumour content deconvolution with an experiment on a small dataset only to showcase its capability. The comparable result with a common method like ISOpure encourages us for more future work in this direction, such as metagenes and sub-network related analysis, as well as content deconvolution.

All methodologies described in this paper are implemented in the R package NNLM, available on CRAN and Github. All the code for the experiments in this paper can be found on the vignette of the package.

## Conclusion

In this work, we generalize the regularization terms in NMF and extend the multiplicative algorithm to the general case. We develop a new solver based on sequential coordinate-wise descent for both KL and MSE losses and demonstrate its efficiency through complexity analysis and numerical experiments. Our method and implementation can also naturally handle missing entries and be used to impute missing values through reconstruction. We show that the NMF imputation method is more efficient and accurate than popular methods. Motivated by the missing value imputation, we introduce a simple and intuitive method to determine the rank of NMF. Finally, by introducing the masking technique, we show that NMF can be applied to tumour content deconvolution and can achieve similar results as compared to existing methods like ISOpure with better computational efficiency.

## Methods

In this section, we generalize the multiplicative algorithms [1] to incorporate regularizations in (3) and briefly argue how they can be derived. We then introduce a new and faster algorithm for NMF with mean square loss (“Alternating non-negative least square (ANLS)” section) and KL-divergence distance loss (“Sequential quadratic approximation for Kullback-Leibler divergence loss” section) with regularizations, based on the alternating scheme. In “Missing entries” section, we address in all algorithms a common problem that some entries of the target matrix may be unreliable or not observed. The method we introduced naturally leads to an intuitive and logically robust method to determine the unknown rank parameter *k* (“Choice of *k*” section) and a novel approach for missing value imputation for array data (“Missing value imputation” section). We then re-examine NMF in “Masking, content deconvolution and designable factorization” section and develop a method to integrate prior knowledge into NMF and guide the decomposition in a more biologically meaningful way, which can be powerful in applications.

### Overview

NMF decomposes a matrix *A* into two matrices with non-negative entries with smaller ranks, *A*≈*WH*, where $A \in \mathbb {R}^{n\times m}, \, W \in \mathbb {R}^{n\times k}, \, H \in \mathbb {R}^{k\times m}$. Without loss of generalization, rows of *A* represent features (e.g. genes, user profiles, etc) and columns of *A* represent samples. Depending on context, *W* can be interpreted as a feature mapping. Rows of *W* represent disease profiles or metagenes [2]. Columns *H* are compact representations of samples, i.e., sample profiles.

Mathematically, NMF can be formulated as an optimization problem as,

1 $$ \min\limits_{W \ge 0, H \ge 0} L(A, WH) + J_{W}(W) + J_{H}(H).  $$

*L*(*x*,*y*) is a loss function, which is mostly chosen to be square error $\frac 12(x-y)^{2}$, or KL divergence distance *x* log(*x*/*y*)−*x*+*y*. The latter can be interpreted as the deviance from a Poisson model.

*J*_*W*_(*W*) and *J*_*H*_(*H*) are regularizations on the *W* and *H* respectively to encourage the desired properties, such as high sparsity, smaller magnitude or better orthogonality. Various regularization forms are introduced [3, 11], but mostly can be unified as the following form,

2 $$ \begin{aligned} J_{W}(W) &= \alpha_{1} J_{1}(W) + \alpha_{2} J_{2}(W) + \alpha_{3} J_{3}(W), \\ J_{H}(H) &= \beta_{1} J_{1}(H^{T}) + \beta_{2} J_{2}(H^{T}) + \beta_{3} J_{3}(H^{T}). \end{aligned}  $$

where

3 $$ \begin{aligned} J_{1}(X) &:= \dfrac12 ||X||_{F}^{2} = \dfrac12 \text{tr}\left(XX^{T}\right)\\ J_{2}(X) &:= \sum\limits_{i < j} (X_{\cdot i})^{T}X_{\cdot j} = \dfrac12 \text{tr}\left(X(E-I)X^{T}\right) \\ J_{3}(X) &:= \sum\limits_{i,j} |x_{ij}| = \text{tr}(XE). \end{aligned}  $$

*I* is an identity matrix, *E* is a matrix of proper dimension with all entries equal to 1, *X*_·*i*_ and *X*_*i*·_ are the *i*^th^ column and row respectively.

*J*_1_ is a ridge penalty to control the magnitudes and smoothness. *J*_1_ also helps stabilize numerical algorithms. *J*_2_(*X*) is used to minimize correlations among columns, i.e., to maximize independence or the angle between *X*_·*i*_, *X*_·*j*_ [11]. *J*_3_ is a LASSO-like penalty that controls matrix-wise sparsity. [3] introduced a different type of regularization to favour sparsity in a column-wise manner as the following,

4 $$ \bar{J} (X) = \dfrac12 \sum\limits_{\bar{k}} ||X_{\bar{k}\cdot}||_{1}^{2} = \dfrac12 \text{tr}\left(XEX^{T}\right).  $$

Obviously, $\bar {J} = J_{1} + J_{2}$, a special case of (2).

Conventionally, (1) is solved by an alternating algorithm, which solves *W* and *H* alternately and iteratively. Thanks to the non-negative constraint, the penalties do not bring additional complexity.

### Adding regularization to Lee’s multiplicative algorithm

Two multiplicative updating algorithms are proposed in [1] for square loss and KL divergence loss. They are adapted by [11] to cases with sparsity regularization. Here we modify these algorithms to integrate all the above regularizations as the following.

With square loss

5 $$ h_{\bar{k}j} \leftarrow h_{\bar{k}j} \frac{(W^{T}A)_{\bar{k}j}}{\left(\left[W^{T}W + \beta_{1} I + \beta_{2} (E-I)\right]H + \beta_{3}E \right)_{\bar{k}j}}.  $$

With Kullback-Leibler divergence distance,

6 $$ h_{\bar{k}j} \leftarrow h_{\bar{k}j} \frac{\sum_{l} \left(w_{l\bar{k}}a_{lj}/\sum_{q}w_{lq} h_{qj}\right)} { \left(\sum_{l} w_{l\bar{k}} + (\beta_{1}-\beta_{2}) h_{\bar{k}j} + \beta_{2} \sum_{l} h_{lj} + \beta_{3} \right) }.  $$

When *β*_*i*_=0, *i*=1,2,3, these are the original multiplicative algorithms in [1]. If *β*_1_=0, these updates reduce to equations (10) and (23) in [11]. The proof when *β*_1_≠0 can be done similarly as in [11]. The updating rules for *W* are similar to (5) and (6).

These multiplicative algorithms are straightforward to implement, but they have the drawback that when an entry of *W* or *H* is initialized as zero or positive, it remains zero or positive throughout the iterations. Therefore, all entries should be initialized to be positive. As a consequence, true sparsity cannot be achieved in general, unless a hard-thresholding is imposed, as many of the entries would be small enough to be thresholded to zero.

### Alternating non-negative least square (ANLS)

When *L* is a square loss, the following sequential coordinate-wise descent (SCD) algorithm proposed by [5] is used to solve a penalized NNLS for *H* while *W* is fixed.

7 $$ \begin{aligned} &\frac{1}{2}||A - WH||_{F}^{2} + J_{H}(H) \\ =\, & \text{tr}\left\{\frac12 H^{T} \left[W^{T}W + \beta_{1} I + \beta_{2} (E-I)\right] H \right.\\&\left.- H^{T}\left[W^{T}A- \beta_{3} E\right]\right\} + const. \end{aligned}  $$

Let

8 $$ \begin{aligned} V =&\, W^{T} W + \beta_{1} I + \beta_{2} (E-I), \\ U =&\, -W^{T}A + \beta_{3} E. \end{aligned}  $$

(7) becomes

9 $$ \begin{aligned} &\text{tr}\left\{\frac12 H^{T} V H + H^{T}U\right\} + const. \\ =\, & \sum_{\bar{k}, j} \left(v_{\bar{k}\bar{k}} h_{\bar{k}j}^{2} + \left(\sum_{l\neq \bar{k}} v_{\bar{k}l} h_{lj} + u_{\bar{k}j}\right) h_{\bar{k}j} \right) \\ \end{aligned}  $$

Since *E*−*I* is semi-negative definite, to ensure the uniqueness and the convergence of the algorithm, we impose the constraint that *β*_1_>*β*_2_, in which case $v_{\bar {k}\bar {k}} > 0$ for all $\bar {k}$.

If all elements of *H* are fixed except for $h_{\bar {k}j}$, then the above is a quadratic function of $h_{\bar {k}j}$ with a non-negative constraint, which can be explicitly optimized by

10 $$ \begin{aligned} h^{*}_{\bar{k}j} =& \max\left(0, -\frac{\sum_{l\neq\bar{k}} v_{\bar{k}l} h_{lj} + u_{\bar{k}j}}{v_{\bar{k}\bar{k}}}\right) \\ =& \max\left(0, h_{\bar{k}j} -\frac{\sum_{l} v_{\bar{k}l} h_{lj} + u_{\bar{k}j}}{v_{\bar{k}\bar{k}}}\right). \end{aligned}  $$

Obviously, *j*=1,…,*m* can be updated independently and in parallel. We then have the following SCD algorithm for solving *H* when *W* is fixed.

1. Initialization. Set
  11 $$  H^{(0)} = 0, \, U^{(0)} = -W^{T}A + \beta_{3} E.  $$
2. Repeat until convergence: for $\bar {k} = 1$ to *k*, update simultaneously and in parallel for *j*=1,…*m*,
  12 $$ {}\begin{aligned} h_{\bar{k}j}^{(t+1)} =& \max\left(0,\,h_{\bar{k}j}^{(t)} - \frac{u_{\bar{k}j}^{(t)}}{v_{\bar{k}\bar{k}}}\right) \text{ and } h_{ij}^{(t+1)} = h_{ij}^{(t)} \,\, \forall i \neq \bar{k}\\ U_{\cdot j}^{(t+1)} =& \begin{cases} U_{\cdot j}^{(t)} - \left(h_{\bar{k}j}^{(t+1)} - h_{\bar{k}j}^{(t)}\right) V_{\cdot\bar{k}}, & \text{if } h_{\bar{k}j}^{(t+1)} \neq h_{\bar{k}j}^{(t)} \\ U_{\cdot j}^{(t)}, & o.w. \end{cases} \end{aligned}  $$
  where $V_{\cdot j}, U_{\cdot j}^{(t)}$ denote the *j*-th column of matrix *V* and $U^{(t)} = \left \{u^{(t)}_{ij}\right \}$.

From (10), one can see that each iteration is non-increasing and therefore the algorithm converges to some fixed point. Any entry of a fixed point should be either on the boundary with its gradient pointing out of the feasible region (*H*≤0) or at a stationary point. A formal proof of convergence can be found in [5].

The alternating algorithm fixes *W* and solves for *H* using NNLS, and then fixes *H* and solves for *W* using the same algorithm. This procedure is repeated until the change of *A*−*WH* is sufficiently small. Each update is non-increasing, thus the alternating algorithm converges.

Instead of initializing *H*^(0)^=0 for every iteration, we use a *warm-start*, i.e., initializing *H*^(0)^ as the result from the previous iteration.

### Sequential quadratic approximation for Kullback-Leibler divergence loss

When *L* is a KL divergence distance, we use a similar SCD algorithm, by approximating KL(*A*|*WH*) with a quadratic function.

Assume *W* is known and *H* is to be solved. Let

13 $$ \begin{aligned} b :=& \frac{\partial \text{KL}}{\partial h_{\bar{k}j}} \left(H^{(t)}\right) = \sum_{l}\left(w_{l\bar{k}} - \frac{a_{lj}w_{l\bar{k}}}{\sum_{q} w_{lq} h_{qj}^{(t)}}\right)\\ a :=& \frac{\partial^{2}\text{KL}}{\partial h_{\bar{k}j}^{2}} \left(H^{(t)}\right) = \sum_{l} a_{lj} \left(\frac{w_{l\bar{k}}}{\sum_{q} w_{iq} h^{(t)}_{qj}}\right)^{2} \end{aligned}  $$

where *H*^(*t*)^ is the current value of *H* in the iterative procedure.

When fixing all other entries, the Taylor expansion of the penalized KL divergence up to the 2nd order at $h^{(t)}_{\bar {k}j}$*w.r.t.*$h_{\bar {k}j}$ is

$${}\begin{aligned} &\, b \left(h_{\bar{k}j} - h^{(t)}_{\bar{k}j}\right) + \frac{a}{2} \left(h_{\bar{k}j} - h^{(t)}_{\bar{k}j}\right)^{2} + \frac{\beta_{1}}{2} h_{\bar{k}j}^{2} \\&+ \beta_{2} \left(\sum_{l\neq \bar{k}} h_{lj}\right)h_{\bar{k}j} + \beta_{3} h_{\bar{k}j} + const \\ =&\, \frac{a + \beta_{1}}{2} h_{\bar{k}j}^{2} - \left(a h^{(t)}_{\bar{k}j} -b - \beta_{2} \sum_{l\neq \bar{k}} h_{lj} - \beta_{3} \right) h_{\bar{k}j} + const. \end{aligned} $$

This can be solved explicitly by

14 $$  h^{(t+1)}_{\bar{k}j} = \max\left(0, \, \frac{a h^{(t)}_{\bar{k}j} - b - \beta_{2} \sum_{l\neq \bar{k}} h^{(t)}_{lj} - \beta_{3}}{a + \beta_{1}} \right).  $$

A similar formula for updating $W_{i\bar {k}}$ can be derived. Note that when an entry of $\hat A = WH$ is 0, the KL divergence is infinity. To avoid this, we add a small number to the denominators in both (13) and (14).

### Complexity and convergence speed

The first step of SCD (Eq. 11) has complexity of *kmn* due to *W*^*T*^*A*. The second step (Eq. 12) costs *km*×*k*×*N*_*i*_, where the second *k* is due to the update of $U_{\cdot j}^{(t+1)}$ and *N*_*i*_ is the number of inner iterations to solve the non-negative linear model. In total, *kmn*+*k*^2^*mN*_*i*_ multiplications are needed for solving *H* given *W* fixed. Accounting the similar computation for *W*, the total complexity of SCD is $\mathcal {O}\left (\left ((m+n)k^{2} N_{i} + 2nmk\right)N_{o}\right)$, where *N*_*o*_ is the number of outer iterations to alternate *W* and *H*. *N*_*i*_*N*_*o*_ is the total number of epochs, i.e., one complete scan over all entries of *W* and *H*. For Lee’s multiplicative algorithm with MSE, when *W* is fixed, the complexity of solving *H* is *knm* for *W*^*T*^*A* on the numerator, *k*^2^*n* for *W*^*T*^*W* on the denominator and *k*^2^*mxN*_*i*_ for multiple *H* at the denominator for *N*_*i*_ times, which add up to *knm*+*k*^2^*n*+*k*^2^*mN*_*i*_. Accounting for *W* and the alternatings, Lee’s algorithm with MSE loss has the same complexity as SCD. The same analysis can be done with their KL counterparts, for which both algorithms have the same complexity of $\mathcal {O}\left (nmk^{2}N_{i}N_{o}\right)$.

Obviously, algorithms with square error loss are faster than the KL based ones (by a factor of *k*) in terms of complexity, and can benefit from multiple inner iterations *N*_*i*_ (reducing the expensive computation of *W*^*T*^*A* and *AH*^*T*^) as typically *k*≪*m*,*n*, which generally should reduce *N*_*o*_. In contrast, algorithms with KL loss cannot benefit from inner iterations due to the re-calculation of *WH* on each inner iteration. Though the SCD and Lee’s algorithm are similar in terms of complexity, one can expect a much faster convergence in SCD. This is because Lee’s algorithm is essentially a gradient descent with a special step size [1] which is a first order method, while SCD is a Newton-Raphson like second order approach.

### Missing entries

Due to various reasons, not all entries of *A* will always present. In some cases, even if an entry is observed, it may not be reliable. In this case, it may be better to treat them as missing entries. Since matrix *A* is mostly assumed to have a low-rank *k*, the information in *A* is redundant for such a decomposition. Hence factorization can be done with the presence of missing entries in *A*, using only the observed ones.

In fact, as the loss function is usually the sum of losses of all elements, it is natural to simply drop losses related to the missing entries. For any *j*, let *I*_*j*_={*i*:*a*_*ij*_ not missing} and $\bar {I}_{j} = \{i: a_{ij} \text { is missing}\}$. When updating the *j*-th column of *H*, all $\bar {I}_{j}$ rows of *W* should be removed, i.e., *U*,*V* in (8) are modified as

15 $$ \begin{aligned} V =&\ W_{I_{j}\cdot}^{T} W_{I_{j}\cdot} + \beta_{1} I + \beta_{2} \,(E-I).\\ U =& \,-W_{I_{j}\cdot}^{T}A_{I_{j}\cdot} + \beta_{3} E, \end{aligned}  $$

where $W_{I_{j}\cdot }$ and $ A_{I_{j}\cdot }$ denote the submatrices of *W* and *A* with row indices in *I*_*j*_. Unlike the non-missing case, *V* depends on *j*.

Similar modification can be applied to the KL counterpart (14) and Lee’s multiplicative algorithms (5, 6) by replacing *W*^*T*^*W* and *W*^*T*^*A* in the same way as in (15). Note that the re-calculation of *V* only increases the complexity of MSE based method but not KL based, in which case it has to be re-computed nevertheless. The ability to handle missing values is crucial in applications, and turns out to induce a novel missing value imputation method (described in “Missing value imputation” section and a novel method for choosing *k* (described in “Choice of *k*” section).

### Missing value imputation

As discussed in “Missing entries” section, the information in *A* is mostly redundant for factorization purposes. Hence reasonable results can still be achieved with missing entries present in matrix *A*. The reconstructions $\hat {A} = WH$ on missing entries are reasonable predictions for the missing values.

The advantage of NMF imputation is that it takes into account all the complete entries when imputing a single missing entry, which implies that NMF can capture complex dependency among entries, while a conventional statistical missing value imputation algorithm, e.g., missForest [9] and MICE [10], usually models missing entries in a feature-by-feature (column-by-column or row-by-row) manner and iterates over all features multiple times to capture complex dependency.

### Choice of *k*

The selection of hyper-parameters is a typical challenge for all unsupervised learning algorithms. The rank *k* is the only but critical parameter, which is a priori unknown. Brunet et al. [2] suggests to try multiple runs of each *k* and uses a consensus matrix to determine *k*. This idea assumes that cluster assignment is stable from run to run if a clustering into *k* classes is strong. However, the assumption needs to be verified and the purpose of NMF is not always for clustering. Besides, the idea of consensus is to choose *k* with lower variation in clustering, which is not necessarily the right measure for choosing *k*. We argue that a reasonable *k* should be able to remove noise and recover the signal. One idea, brought from the denoising auto-encoder [17], is to add noise to the matrix *A*, factorize the noisy version and compare the reconstructed matrix to the original *A*. One can expect that the “correct" *k* should give the smallest error rate. This could be a general approach for many unsupervised learning algorithms. However, when it comes to NMF, the choice of noise is not obvious as the noisy version of *A* has to be non-negative as well, which suggests that injected noise may also introduce bias. In addition, the choice of the noise distribution is yet another hyperparameter not obvious to pick.

Given the ability to handle missing entries in NMF described in the above section and the powerful missing value imputation of NMF demonstrated in “Missing value imputation” section, we come up with a novel approach, akin to the well-known the training-validation split approach in supervised learning.

1. Some portion (e.g., 30%) of entries are randomly deleted (selected to be missing) from *A*.
2. The deleted entries are imputed by NMF with a set of different *k*’s.
3. The imputed entries are compared to their observed values, and the *k* that gives the smallest error is selected.

The above approach can be argued by the assumption that only the correct *k*, if exists, has the right decomposition that can recover the missing entries. In contrast to the training-validation split in supervised learning, due to the typically big number of entries in *A*, we generally have a very large ‘sample size’. One can also easily adapt the idea of cross-validation to this approach. This idea should apply to any unsupervised learning method that handles missing values. Note that bootstrapping and cross-validation can also be easily incorporated here.

### Masking, content deconvolution and designable factorization

Microarrays are popular techniques for measuring mRNA expression. Strictly speaking, an mRNA profile of a certain tumour sample is typically a mixture of cancerous and healthy profiles as the collected tissues are ‘contaminated’ by healthy cells. A pure cancer profile is usually more suitable for downstream analysis [14].

One can utilize NMF for such a purpose by formatting it as

16 $$ A \approx W H + W_{0} H_{1},  $$

where matrix *W* is an unknown cancer profile, and matrix *W*_0_ is a known healthy profile. Rows of *A* represent probes or genes while columns represent patients or samples. The task here is to solve *W*, *H* and *H*_1_ given *A* and *W*_0_, which can be thought of as a ‘guided’ NMF. In this decomposition, the number of columns of *W* can be interpreted as the number of unknown cancerous profiles or cell types. The corresponding tumour percentage of sample *j* can be estimated as

17 $$ \hat r_{j} = \frac{\sum_{i} W_{i, \cdot} H_{\cdot, j}} {\sum_{i} \left(W_{i \cdot} H_{\cdot j} + W_{0, i\cdot} H_{1, \cdot j}\right)}.  $$

A more general implementation is to use mask matrices for *W* and *H*, where the masked entries are fixed to their initial values or 0 if not initialized. Indeed, one can treat this as a form of hard regularization. It can be seen immediately that the above deconvolution is a special case of this masking technique, in which the masked entries are initialized to the known profile and fixed. This technique is designed to integrate domain knowledge, such as gene sub-networks, pathways, etc, to guide the NMF towards a more biologically meaningful decomposition.

For example, assume $\mathcal {S} = \{S_{1},..., S_{L}\}$, where each *S*_*l*_, *l*=1,...,*L* is a set of genes in certain sub-network or pathway *l*. One can design *W* as a matrix of *K* columns (*K*≥*L*), with *w*_*il*_=0 when *i*∉*S*_*l*_. NMF factorization will learn the *weight* or *contribution* *w*_*il*_ of real gene *i* in sub-network or pathway *l* from data. One can also interpret $ h_{l j}\left (\sum _{i} w_{i l}\right) $ as an expression level of sub-network or pathway *l* in patient *j*. Besides, *W*_·*j*_’s for *j*=*L*+1,…,*K* are unknown sub-networks or pathways. Note that *K* is unknown beforehand, but can be determined by the method introduced in “Choice of *k*” section.

Similarly, if *S*_*l*_ is a set of marker genes (those that are known to be expressed only in a specific cell type / tissue), for tissue *l*, by letting $w_{i l} = 0, \, i \in \bigcup \limits _{q\neq l} S_{q}$, one can find the relative abundance of each tissue type in a sample. A similar formula to Eq. (17) can be used to compute the proportions of known (*j*=1,…,*L*) and unknown (*j*=*L*+1,…,*K*) cell types / tissues.

Another possible application of masking is meta-analysis of different cancer types which finds metagenes that are shared among cancers. For instance, assume *A*_1_,*A*_2_ are expressions of lung cancer and prostate cancer microarrays. By setting certain parts of the coefficient matrix *H* to 0, for example,

18 $$ \left(A_{1}\,A_{2}\right) = \left(W_{0}\,W_{1}\,W_{2}\right) \left(\begin{array}{cc} H_{01} & H_{02} \\ H_{1} & 0\\ 0 & H_{2} \end{array}\right),  $$

we can expect that *W*_1_ and *W*_2_ are lung and prostate cancer specific profiles, while *W*_0_ is a shared profile.

## Data Availability

The subset of Botling’s NSCLC dataset [8] used in the paper is available in R package NNLM. Code for all experiments can also be found in the vignette at https://cran.r-project.org/web/packages/NNLM/vignettes/Fast-And-Versatile-NMF.pdf. The package NNLM is available on CRAN and on https://github.com/linxihui/NNLM.

## Abbreviations

ANLS: Alternating non-negative least square
KL divergence: Kullback–Leibler divergence
MKL: Mean KL divergence
MSE: Mean square error
NMF/NNMF: Non-negative matrix factorization
NNLM: Non-negative linear model
NNLS: Non-negative least square
SCD: Sequential coordinate-wise descent
